# Mild traumatic brain injury increases engagement in criminal behaviour 10 years later: a case–control study

**DOI:** 10.3389/fpsyt.2023.1154707

**Published:** 2023-05-02

**Authors:** Alice Theadom, Lisa Meehan, Sandra McCallum, Gail Pacheco

**Affiliations:** ^1^Traumatic Brain Injury Network, Auckland University of Technology, Auckland, New Zealand; ^2^New Zealand Work Research Institute, Auckland University of Technology, Auckland, New Zealand

**Keywords:** traumatic brain injury, mTBI, concussion, criminal behaviour, behaviour, violence, conviction

## Abstract

**Introduction:**

Sustaining a mild traumatic brain injury (mTBI) has been linked to increased criminal behaviour in later life. However, previous studies have not controlled for the number of injuries, gender, social deprivation, impact of past behaviour, or link to offence type. This study aims to determine if people who experienced a single or multiple mTBI have increased risk of criminal behaviour 10 years post-injury than matched orthopaedic controls.

**Methods:**

This was a case control study of adults (aged >16 years) who experienced a medically diagnosed mTBI and controls who experienced a lower limb fracture (with no TBI) over a 12-month period (01/01/2003–31/12/2003). Participants were identified within Stats New Zealand’s Integrated Data Infrastructure (national database including health and justice records). Participants who experienced a subsequent TBI (post-2003), who were not resident in New Zealand, and who died by 2013 were excluded. Case and controls were matched by age, sex, ethnicity, deprivation index and pre-injury criminal history.

**Results:**

The study included *N* = 6,606 mTBI cases and *N* = 15,771 matched trauma controls. In the 10 years after injury, people experiencing a single mTBI had significantly higher numbers of violent charges (0.26 versus 0.21, *p* < 0.01) and violent convictions (0.16 versus 0.13, *p* < 0.05) but not for all court charges and convictions. Analysis of those with a history of prior mTBIs yielded larger effects, with significantly higher numbers of violent charges (0.57 versus 0.24, *p* < 0.05) and violent convictions (0.34 versus 0.14, *p* < 0.05). For males, the single mTBI case group had a significantly higher number of violent charges (0.40 versus 0.31, *p* < 0.05) and violent convictions (0.24 versus 0.20, *p* < 0.05) but this was not observed for females or all offence types.

**Discussion:**

Experiencing multiple mTBIs over the lifetime increases the number of subsequent violence-related charges and convictions but not for all offence types in males but not for females. These findings highlight the need for improved recognition and treatment of mTBI to prevent future engagement in antisocial behaviour.

## 1. Introduction

There is increasing global concern regarding the possible longer-term effects of mild traumatic brain injuries (mTBI, including concussion) (1) This is of concern as up to 90% of traumatic brain injuries are classified as mild in severity (2). Whilst many recover well after mTBI, nearly a third of people endure chronic persistent symptoms and disability (3). Symptoms can include headaches, irritability, difficulties sustaining attention or remembering information, sensory sensitivities (e.g., sensitivity to noise and light), dizziness and fatigue (4). Additionally, there is evidence that there may be cumulative effects (increased symptoms) from repeated mTBIs sustained over the lifetime (5). Persistent impacts of mTBI can include reduced self-confidence, coping strategies and social integration making it harder to be successful at school, in employment or function in everyday life and may increase the risk of engagement in antisocial behaviour (6, 7).

Longitudinal studies have revealed that sustaining a mTBI in childhood or early adolescence is linked to an increased risk of engagement in violence, being drunk and disorderly, fines and involvement in petty crime compared to orthopaedic controls (8). Additionally, a longitudinal study of young adults who had sustained a TBI found that those who had experienced a least one prior TBI were more likely to be incarcerated than those without a prior TBI, supporting potential wider cumulative effects (9). However, the link between TBI and longer term behaviour has not always been consistent. For example, a study based on self-reported TBI (of any severity) during childhood found that the TBI group reported increased trouble with the police and more parent-reported conduct problems than non-injured controls, but they found no difference in comparison to orthopaedic controls (10). This may suggest that the trauma of experiencing an injury of any type may influence longer-term behaviour more than the type of injury or conversely that children with behavioural issues may be more likely to sustain a TBI. Consequently, use of orthopaedic controls may therefore assist in controlling for potential behavioural differences in these populations to determine any specific effects due to mTBI.

Few studies of the potential longer-term impacts of TBI sustained in adults on behaviour in the general population have been conducted. One study in Australia, revealed that adults who were admitted to hospital for a TBI over a five-year timespan had increased rates of criminal conviction in comparison to uninjured siblings and controls who had never been admitted to hospital (11). This latter study enabled the control of familial factors such as ethnicity and socioeconomic status, however included moderate and severe TBI making it difficult to determine any specific effects of mTBI. It is likely that the link between mTBI and criminal behaviour is also likely to be highly complex (12). For example, sociodemographic variables such as age of injury, social deprivation, sex and pre-injury criminal behaviour are all likely to influence the relationship. To enable better understanding of the link between mTBI and criminal behaviour, these potential sociodemoegraphic factors need to be controlled for to determine any likely specific effect of mTBI on criminal behaviour in later life (13, 14).

This study aims to determine if there is a link between single and multiple mTBI histories and criminal charges and convictions over the 10 year period post injury compared with orthopaedic controls, controlling for age, gender, ethnicity, socioeconomic status and previous criminal charges.

## 2. Materials and methods

### 2.1. Participants

This was a case control study comprising of patients aged >16 years at time of injury (the age at which youth enter the adult justice system in New Zealand (NZ).

Data were extracted from the Integrated Data Infrastructure (IDI) database which links national administrative data across government agencies *via* a unique identifier. Key data sources for this study include injury data extracted from the Accident Compensation Corporation (ACC, a national insurance provider who provides no-fault health cover for all injuries, reducing the risk of underreporting and misreporting) and the Ministry of Justice. The use of ACC data enabled the inclusion of mTBI cases receiving medical treatment who attended hospital as well as primary care settings (e.g., general practitioners) (15). This may be particularly pertinent in the context of mTBI where individuals may be more likely to seek medical treatment *via* primary care providers (16).

To be included in the analysis as a case or control, participants needed to be >16 years at the time of the index injury and classified as a NZ resident (defined as being present in NZ 75% or more of the time in the 10 years from their injury date based on Ministry of Business, Innovation and Employment border movements data). These inclusion criteria aimed to ensure a period of 10 years of follow up data was available in the adult justice system.

mTBI cases were identified based on those who had a medically diagnosed mTBI claim in the ACC database between 01/01/2003 and 31/12/2003 based on ICD-9 code 850 and ICD-10 code S06.0 and/or ACC READ code S6 (ACC READ code S6 is an organisation specific code used to indicate the diagnosis of concussion by a medical practitioner). The index injury refers to the first mTBI experienced in the study case registration timeframe. Cases were excluded if they experienced a subsequent TBI of any severity in the 10 year follow up period (until 31/12/2013) or if they had experienced a moderate or severe TBI prior to their index mTBI in 2003. Exclusions were applied to exclude any recency effects from subsequent injuries or skewed results following inclusion of more severe TBIs. However, cases who had sustained mTBI prior to 2003 were retained and analysed separately to explore potential impacts from single and multiple mTBI impacts on engagement in criminal behaviour.

The control group included people who experienced a lower limb fracture between 01/01/2003 and 31/12/2003 in 2003 based on ICD 9 codes 820–829 and ICD 10 codes S72, S82 and S92 and ACC READ code S3, at the time of accident. Lower limb fracture controls were selected to account for the possible impact of accident trauma on outcomes but reduced the risk of a missed TBI diagnosis than if upper limb injuries had been used (17). Age and gender were extracted from the Stats NZ’s personal details database. Self-reported ethnicity was extracted from the ACC data. A deprivation index (as a measure of socioeconomic status) was based on the meshblock (a statistical area approximately equivalent to a city block for urban areas) the person lived in the most days during 2003 (at the time of injury) (18).

### 2.2. Outcome measures

Data from the Ministry of Justice on the number and offence type of court charges (criminal charges placed against a person but not necessarily resulting in a conviction) and criminal convictions were extracted. All charges and convictions as well the subset of charges/convictions relating to violent offences were examined. Violent offences are classified in the data by the Ministry of Justice and include offences such as murder, attempted murder, manslaughter, abduction, assault and aggravated robbery.

### 2.3. Propensity score matching

To enhance comparability of the groups (as the mTBI group is more likely to be male, of non-European ethnicity and of a younger age based on incidence data) (16), propensity score matching was used. Cases and controls were matched on age, gender, ethnicity, deprivation index and the number of previous offending incidents. Logistic regressions with a caliper match of 0.06 were used. Conditions for propensity matching such as overlapping support were met. All those in the mTBI treatment group were successfully matched with at least one person in the control group (mean number of controls to cases was 2.4).

### 2.4. Statistical analysis

The case and controls were compared *via* estimation of the average treatment effect on the treated, where treatment was experiencing a single mTBI in 2003. Criminal behaviour outcomes examined included the number of court charges and convictions for any offence type. Analysis was then specifically conducted on court charges and convictions related to violent offences and by gender. Additional analysis was also conducted for those with previous mTBIs prior to the 2003 injury (i.e., from 1994 when ACC records begin) in order to assess if these have a cumulative effect on criminal behaviour outcomes. This involved applying the additional matching criterion of the number of previous mTBIs for this sub-analysis. To determine if group differences remained, distributions of the propensity scores and the standardised differences and variance ratios of the case and control group were examined.

## 3. Results

**Figure 1:**
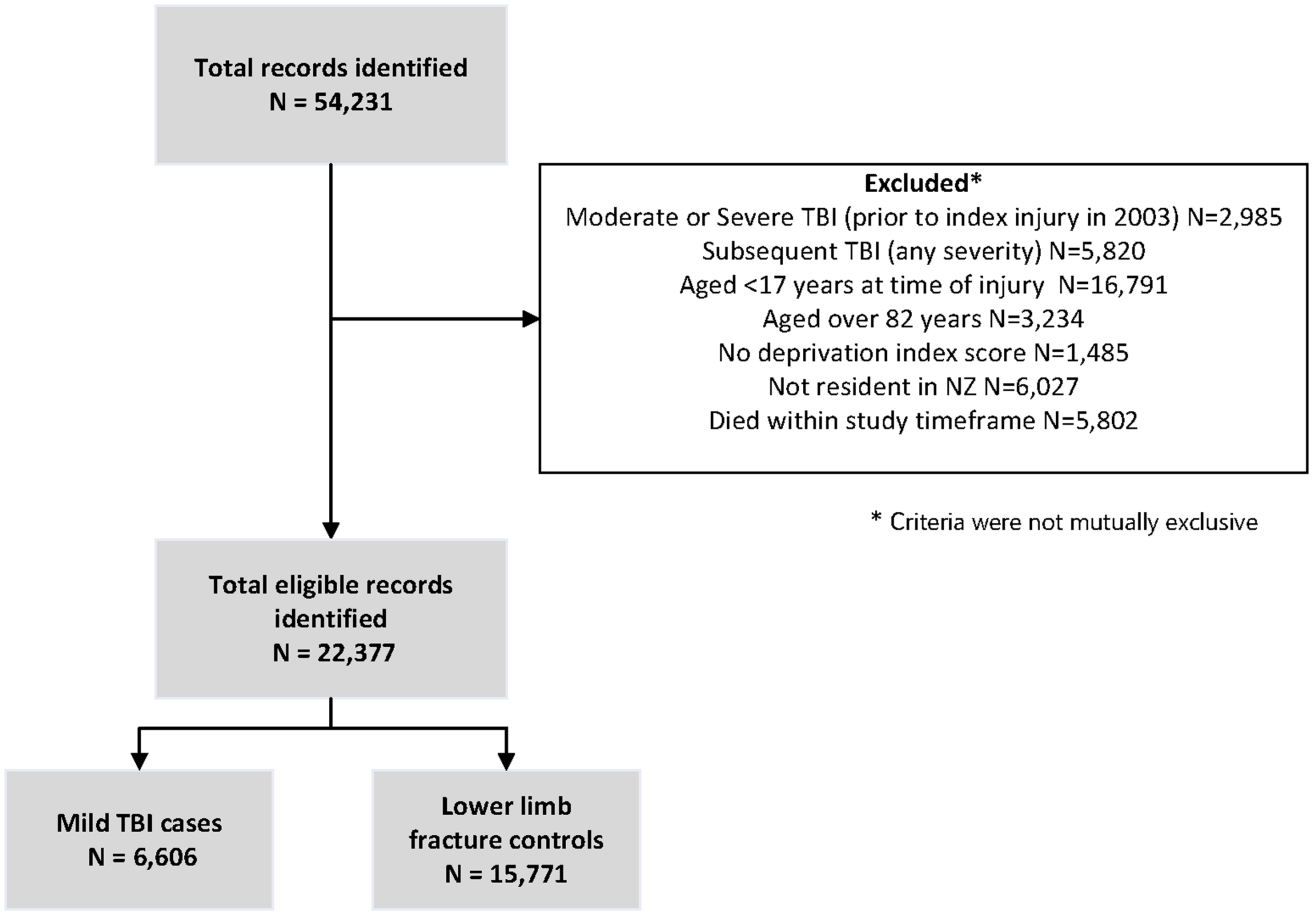
Selection of participants.

Selection of participants for this analysis based on the inclusion criteria is outlined in Figure 1. There were *N* = 6,606 cases, and following propensity score matching, *N* = 15,771 controls.

**Figure 2:**
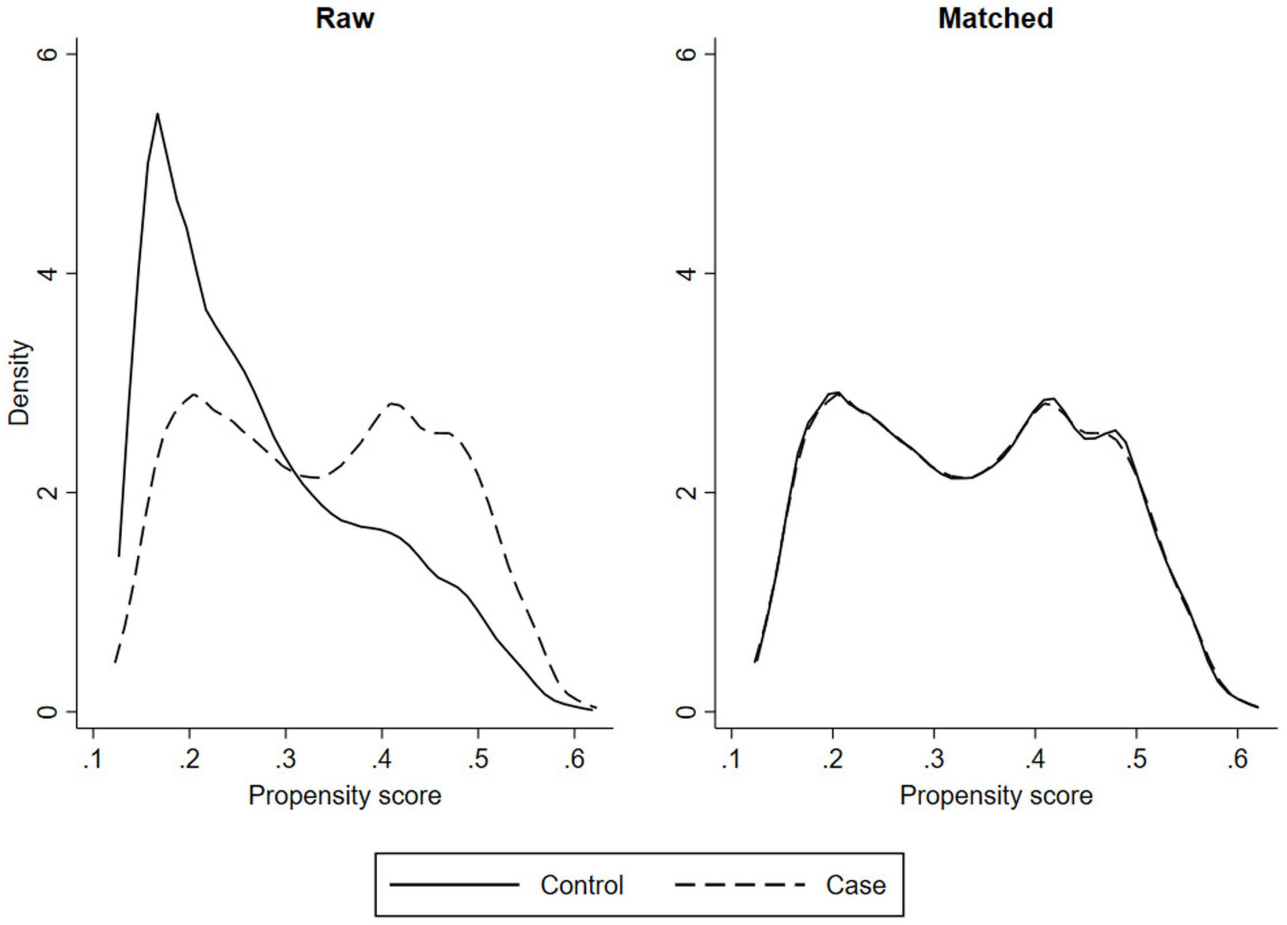
Non-parametric density plots.

Figure 2 uses non-parametric kernel density plots to show the distribution of the propensity scores – i.e. the probability that an individual will be in the case group based on the observed characteristics (age, gender, ethnicity, deprivation index and number of previous offending incidents). The right-hand panel shows that before matching, the distributions of the case and control groups are very different. The left-hand panel shows that after matching, the distributions of the case and control groups are virtually identical. Comparing the means, the absolute values of the standardised difference between case and control groups for the matched samples were close to zero (maximum value of 0.04; Supplementary Table S1). Comparing the variances, the variance ratios were close to 1 (maximum value of 1.05; Table 1) (19). Likewise, for the sample of males only (not shown), the propensity score distributions of the case and control groups were similar, with the maximum absolute standardised difference of 0.04 and the maximum variance ratio of 1.04. The sample of females only was less well matched on the previous court charges variable (standardised difference of 0.08 and variance ratio of 1.17), but well matched on the other variables. For the sample of those with mTBIs before 2003, the matching on European, deprivation level and number of previous mTBIs is less well matched (maximum variance ratio of 1.2 for number of previous mTBIs), likely due to the much smaller sample size.

**Table 1:** Criminal behaviour outcomes between single mTBI cases and the orthopedic control group.

	Mean: total	Mean: single Mild TBI cases	Mean: control group	Coefficient (Standard deviation)	*p*-value
No previous TBI	*N* = 22,377	*N* = 6,606	*N* = 15,771	–	–
Number of charges for any offence	1.63	1.71	1.56	0.15 (0.11)	0.17
Number of convictions for any offence	1.22	1.27	1.16	0.11 (0.08)	0.18
Number of court charges for violent offences	0.23	0.26	0.21	0.05 (0.02)	0.01
Number of convictions for violent offences	0.14	0.16	0.13	0.03 (0.01)	0.03

The effect of a single mTBI event is shown in Table 1, which restricts the sample to those who had not experienced a previous mTBI. Effects were estimated for the number of overall and violent charges and convictions over a 10 year post-injury period. Table 1 excludes those with a prior TBI history in order to examine the effect of a single mTBI. The number of charges for any offence type (1.71 versus 1.56) and the number of convictions for any offence type (1.27 versus 1.16) was higher among the mTBI group. However, these differences were not statistically significant. In terms of violent offending, the number of violence-related charges (0.26 versus 0.21) and convictions (0.16 versus 0.13) was higher among the mTBI group, and the difference statistically significant. For those with single mTBI in 2003, 28% had at least one court charge prior to 2003 injury. For those with previous mTBIs, 35% had at least one court charge prior to the 2003 injury.

Table 2 presents results for the sample who experienced previous mTBIs prior to 2003. Comparing Tables 1, 2 shows that the number of overall and violence-related charges and convictions was 1.6–1.7 times higher among the sample with previous mTBIs compared with the sample with a single mTBI. As with the single mTBI sample, there is no statistically significant difference between the number of offences and convictions for any type of offence between the case and control groups. However, there was a significant difference in the number of charges (0.57 versus 0.24) and convictions (0.34 versus 0.14) for violent offences. Moreover, the magnitude of this effect was larger for this sample with previous mTBIs than for the sample with a single mTBI.

**Table 2:** Criminal behaviour outcomes between multiple mTBI cases and the orthopedic control group with a TBI history.

	Mean: total	Mean: multiple Mild TBI cases	Mean: control group	Coefficient (Standard deviation)	*p*-value
Previous mTBI	*N* = 759	*N* = 414	*N* = 345		
Number of charges for any type of offence	2.57	3.07	2.06	1.01 (0.66)	0.13
Number of convictions for any type of offence	1.93	2.31	1.55	0.76 (0.5)	0.13
Number of court charges for violent offences	0.40	0.57	0.24	0.32 (0.14)	0.02
Number of convictions for violent offences	0.24	0.34	0.14	0.19 (0.08)	0.01

Analysis by gender was undertaken for the sample with no previous TBI since the number of court charges and convictions were more than 4 times higher for men than women (Table 3). The total number of court charges (2.50 versus 2.35) and convictions (1.90 versus 1.77) were higher among the male mTBI case group compared with the control group over the 10 year post-injury period, but the differences are not statistically significant. However, the number of court charges for violent offences (0.40 versus 0.31) and convictions for violent offences (0.24 versus 0.20) is higher among the mTBI group and statistically significant for men.

**Table 3:** Criminal behaviour outcomes by gender excluding prior TBI.

	Mean: total	Mean: single Mild TBI cases	Mean: control group	Coefficient (Standard deviation)	*p*-value
Male	*N* = 11,037	*N* = 3,819	*N* = 7,218		
Number of charges for any offence type	2.43	2.50	2.35	0.17 (0.18)	0.34
Number of convictions for any offence type	1.83	1.90	1.77	0.11 (0.14)	0.41
Number of court charges for violent offences	0.36	0.40	0.31	0.09 (0.04)	0.01
Number of convictions for violent offences	0.22	0.24	0.20	0.04 (0.02)	0.05
**Female**	***N* = 11,340**	***N* = 2,790**	***N* = 8,550**		
Number of charges for any offence type	0.56	0.61	0.51	0.09 (0.09)	0.29
Number of convictions for any offence type	0.40	0.44	0.35	0.08 (0.07)	0.21
Number of court charges for violent offences	0.07	0.07	0.07	0.003 (0.02)	0.85
Number of convictions for violent offences	0.04	0.04	0.04	0.001 (0.01)	0.92

For women with no previous TBIs, there was no observed effect of a single mTBI. The number of court charges (0.61 versus 0.51) and convictions (0.44 versus 0.35) for any offence type is higher among the mTBI case group compared with the control group, but the differences were not statistically significant. There was no difference in the number of court charges and convictions relating to violent offences.

## 4. Discussion

This study aimed to determine if people who experienced a single or multiple mTBI had increased risk of criminal behaviour 10 years post-injury than matched orthopedic controls. It was revealed that people who experienced a single mTBI have a higher number of violent charges and convictions over the following 10 year post-injury period, e.g. but this was only observed violent offences. The link between mTBI and number of criminal convictions and court charges was stronger for those with a prior TBI history, suggesting a cumulative effect. The results hold most strongly for men, with the effect of a single mTBI on criminal activity of women not observed.

The link between mTBI and increased engagement in criminal activity augments previous studies exploring the impacts of mTBI in adolescence and young adulthood (8, 9), and TBI of all severities in adults (11, 20) by revealing that the effects of mTBI sustained in adulthood is linked to increased risk of criminal behaviour in the longer-term. Our findings strengthen the existing evidence as the link remained despite our sample being inclusive of mTBIs that presented to their GP rather than to hospital (indicative of milder acute impact on the person). The findings also provide evidence that the link between TBI and violent criminal behaviour remains after controlling for known predictors of criminal behaviour such as deprivation, prior criminal behaviour, ethnicity and age.

The effect of a single mTBI on criminal activity over 10 years was smaller than the effect observed in those with a prior history of mTBI. This finding augments evidence from the sports context that the effects of mTBI can be cumulative, extending from an increase in persistent symptoms and changes in brain structure to behaviour (21). Previous studies have shown that persistent symptoms can impact on daily functioning and maintenance of employment in the longer term (22, 23). Consequently, it may be likely there is a similar link between persistent mTBI symptoms and criminal behaviour. However, we were not able to determine from the data used in the study the prevalence and type of persistent symptoms and how this impacted the relationship between mTBI and criminal behaviour. Future prospective studies are needed to determine the relative contributions of mTBI, persistent symptoms whilst controlling for sociodemographic and lifestyle factors on behaviour in the longer term. The findings suggest that there is the potential that improved diagnosis and treatment of mTBI symptoms could assist in the prevention of violent criminal behaviour in the longer term.

The significantly increased risk of engagement in criminal behaviour following mTBI in males but not females when analysed separately is of note. In a previous longitudinal study of all severities of TBI in adults, the link was found in both males and females when using general population controls, although no longer remained significant when sibling controls were used (11). This finding may reflect that men are more likely to be arrested for criminal activities than females (20) and additionally have a higher risk of TBI (24). Extraction of the data over a period of 10 years since the date of the incident mTBI date enabled the control of data collected over a controlled time period since the injury. However, this methodology prevented the exploration of the potential link between engagement in criminal activity and increased risk of TBI. A review of the evidence between mTBI and criminal behaviour proposed that the links between mTBI and criminal behaviour are likely to be bidirectional (12). Indeed violence is the third most common cause of TBI (2). To try and address the study did control for prior engagement in criminal behaviour and social deprivation and through its use of orthopaedic controls to account for risk of being injury prone.

There were some limitations of the study. For example, mTBI cases only included people who had experience mTBIs that had been medically diagnosed. There is evidence that many people do not seek medical treatment following a mTBI (16) due to restricted access to transport, lack of awareness, cost or fear of repercussion (25). Consequently many mTBIs where people did not present for medical treatment, particularly within vulnerable population groups are likely to have been missed and the true effect may have been underestimated. The codes used to extract the mTBI cases also only included use of a limited number of injury codes to ensure specificity to mTBI, it is likely that some mTBI cases were coded using alternative codes and were not included in the analysis and some controls may have experienced a mTBI. Additionally, codes were only available at the highest level, e.g., S06.0 in most cases and so we not able to determine if some people with prolonged loss of consciousness of more than 30 min (indicative or a more severe TBI) were included in the analysis. Whilst some factors that can influence engagement in criminal behaviour such as age, gender, deprivation and prior behaviour were accounted for, the study was not able to account for the potential impact of alcohol and substance use and mental health which may be potential influencing factors. We were also not able to explore potential links between early criminal behaviour and risk of TBI which would be important to explore in future research. Despite these limitations this study has revealed initial evidence supporting a link between mTBI and later life violent criminal behaviour.

## Data availability statement

The original contributions presented in the study are included in the article/Supplementary material, further inquiries can be directed to the corresponding author.

## Ethics statement

The studies involving human participants were reviewed and approved by Integrated data infrastructure review committee. Written informed consent for participation was not required for this study in accordance with the national legislation and the institutional requirements.

## Author contributions

AT, GP, and LM designed the study. AT, GP, LM, and SM conducted the study, including data extraction. LM and SM contributed to the data analysis. AT prepared the manuscript draft, with important intellectual input from GP, LM and SM. All authors contributed to the article and approved the submitted version.

## Funding

The study was supported by a Rutherford Discovery Fellowship administered by The Royal Society – Te Apārangi.

## Conflict of interest

The authors declare that the research was conducted in the absence of any commercial or financial relationships that could be construed as a potential conflict of interest.

## Publisher’s note

All claims expressed in this article are solely those of the authors and do not necessarily represent those of their affiliated organizations, or those of the publisher, the editors and the reviewers. Any product that may be evaluated in this article, or claim that may be made by its manufacturer, is not guaranteed or endorsed by the publisher.
