# Supplementary material for: Mild traumatic brain injury increases engagement in criminal behaviour 10 years later: a case–control study
Source: Front Psychiatry. 2023 May 2;14:1154707. doi: 10.3389/fpsyt.2023.1154707 (PMC10197901; doi:10.3389/fpsyt.2023.1154707)
Supplement: Supplementary file 1 [file Table_1.DOCX]

Supplementary Material

Mild traumatic brain injury increases engagement in criminal behaviour 10 years later: A case-control study

Alice Theadom^1*^, Lisa Meehan^2^, Sandra McCallum^1,2^ Gail Pacheco^2^

*** Correspondence:**Alice Theadom
alice.theadom@aut.ac.nz

**Supplementary Table 1. Propensity score matching balance: Standardised differences and variance ratios**

|  | Standardised difference | | Variance ratio | |
| --- | --- | --- | --- | --- |
|  | Raw | Matched | Raw | Matched |
| Female | -0.24 | 0.01 | 0.98 | 1.00 |
| Age at 2003 injury | -0.52 | 0.01 | 0.85 | 1.03 |
| Age squared | -0.48 | 0.02 | 0.70 | 1.03 |
| European | -0.17 | -0.03 | 1.21 | 1.03 |
| Deprivation index | 0.11 | -0.01 | 1.02 | 1.00 |
| Any court charge before 2003 injury | -0.23 | 0.04 | 1.34 | 1.05 |

Notes: Deprivation index is measured on a 10-point scale where 10 is the most deprived and 1 is the least deprived
